# Skin Histology of *Pithecopus* spp. and Modulation of Glutathione Status by the Antioxidant Tryptophyllin PaT-2

**DOI:** 10.3390/antiox15091087

**Published:** 2026-08-29

**Authors:** Flávia G. D. Araújo, Henrique L. L. de Araújo, Maria da Gloria da Silva, João B. Nunes, João G. T. L. Resende, Fernanda L. Silva, Miguel G. Cardoso, Eder A. Barbosa, Andreanne G. Vasconcelos, Guilherme D. Brand, Tatiana K. S. Borges, Amilcar S. Damazo, Daniel C. Moreira, José R. S. A. Leite

**Affiliations:** 1 Applied Immunology and Morphology Research Centre (NuPMIA), Morphology Area, Faculty of Medicine, University of Brasília (UnB), Brasília 70910-900, DF, Brazil; 2 Laboratory of Nanoscience and Nanobiotechnology (LNN), Institute of Biology (IB), University of Brasília (UnB), Brasília 70910-900, DF, Brazil; 3 Laboratório de Síntese e Análise de Biomoléculas (LSAB), Instituto de Química (IQ), Universidade de Brasília (UnB), Brasília 70910-900, DF, Brazil; 4 Faculdade de Farmácia, Universidade de Lisboa, 1649-003 Lisboa, Portugal; 5 People&Science Pesquisa Desenvolvimento e Inovação Ltda, Centro de Apoio ao Desenvolvimento Tecnológico (CDT), Universidade de Brasília (UnB), Brasília 70910-000, DF, Brazil; 6 The Bridge, University of Lincoln, Lincoln LN6 7EL, UK; 7 Laboratório de Imunologia Celular (LIC), Núcleo de Pesquisa em Morfologia e Imunologia Aplicada, (NuPMIA), Faculdade de Medicina (FM), Universidade de Brasília (UnB), Brasília 70910-900, DF, Brazil

**Keywords:** Phyllomedusidae, oxidative stress, MALDI imaging mass spectrometry, cutaneous glands, microglia, menadione

## Abstract

Amphibians of the family Phyllomedusidae produce complex cutaneous secretions with ecological and pharmacological relevance. Here, we investigated the skin morphology of *Pithecopus oreades* and *P. azureus* and the antioxidant function of the tryptophyllin PaT-2 in microglial cells. Histological and histochemical analyses revealed a conserved glandular organization, with mucous glands rich in neutral mucopolysaccharides and serous glands containing acidic and sulfated polysaccharides, concentrated predominantly in the dorsal and cephalic regions. MALDI mass spectrometry imaging showed that PaT-2 (*m*/*z* 696.4) is heterogeneously distributed. A second ion (*m*/*z* 1626.8), assigned to a truncated form of a phylloseptin-like antimicrobial peptide, was predominantly localized in limb tissues and partially overlapped with PaT-2, suggesting co-deployment of antioxidant and antimicrobial molecules on the skin. In BV2 microglial cells, menadione (10 µM) induced oxidative stress reflected by increased oxidized glutathione (GSSG) and higher total glutathione levels, consistent with a compensatory increase in glutathione synthesis. Synthetic PaT-2 (50 µM) was associated with numerically lower GSSG accumulation and a smaller increase in total glutathione when co-administered with menadione, showing no cytotoxicity while demonstrating modulation of endogenous glutathione status. These findings advance the integumentary biology of *Pithecopus*, suggest differences in peptide distribution between the specimens examined, and extend the antioxidant characterization of PaT-2 to the level of glutathione status, supporting its further investigation as a candidate molecule for oxidative stress-related research.

## 1. Introduction

The Phyllomedusidae family, commonly known as leaf frogs, comprises some of the most pharmacologically relevant amphibians known. These arboreal frogs undergo integumentary specializations during metamorphosis that are critical for osmoregulation, respiration, and protection in the terrestrial environment [1,2]. Histologically, their skin consists of an epidermis and dermis, the latter housing specialized mucous and serous (granular) glands whose secretions mediate diverse physiological and ecological functions. Mucous glands produce a glycoprotein-rich fluid essential for hydration and skin flexibility, while serous glands release bioactive molecules—including toxins, antimicrobial peptides, and antioxidant compounds—that constitute the primary chemical defense of these animals [3,4,5].

**Figure 1:**
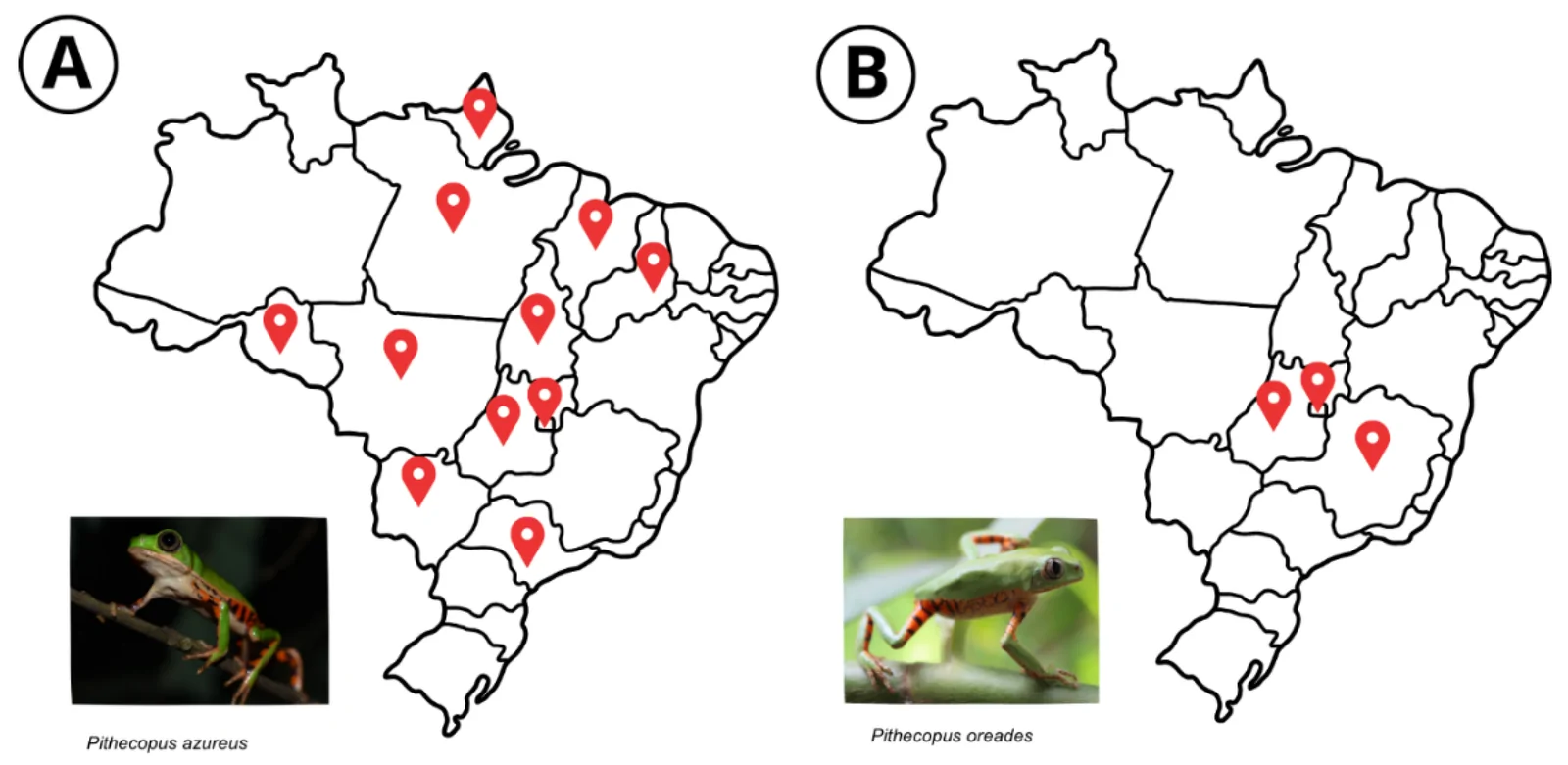
Distribution map of *Pithecopus azureus* and *P. oreades*. (**A**) States of occurrence of *P. azureus* in Brazil; (**B**) occurrence of *P. oreades* [2] (Photos: (**A**): Miguel G. Cardoso; (**B**): Jessica L. S. Costa).

Two species within the genus *Pithecopus* are the focus of this study (Figure 1). *Pithecopus oreades* is endemic to Brazil, restricted to high-altitude areas (~900 m) within the Cerrado biome, with occurrence in the states of Goiás, Minas Gerais, and the Federal District. *Pithecopus azureus* has a broader distribution, spanning eastern Colombia, Venezuela, the Guianas, and the Brazilian Amazon (Pará and Amapá), as well as parts of Mato Grosso, Goiás, and Tocantins [6]. In addition to deterring predators, their cutaneous secretions limit microbial proliferation on the moist integument, protecting the skin from opportunistic infections [3,4]. Despite their distinct biogeographical ranges, both species share the glandular integumentary organization typical of the Phyllomedusidae, yet their comparative skin morphology remains poorly characterized.

Among the bioactive compounds secreted by Phyllomedusinae amphibians, tryptophyllins constitute a structurally diverse class of short peptides characterized by the presence of tryptophan residues in distinct sequence contexts [7]. First described in *Phyllomedusa rohdei* [7], more than 40 variants have since been identified in amphibian skin secretions [8]. Despite this diversity, their biological functions remain incompletely understood, with available evidence pointing to potential antimicrobial, neuromodulatory, and antioxidant activities [3,9]. The amphibian integument is continuously subjected to oxidative challenges—including UV radiation, microbial colonization, and environmental pollutants—and antioxidant peptides present in the skin secretome are thought to represent an additional layer of defense, complementing the canonical enzymatic and glutathione-based endogenous antioxidant systems [10].

One member of this class, the tryptophyllin PaT-2 (FPPWL-NH_2_), has emerged as a promising antioxidant candidate. Originally identified in our previous work in *Pithecopus azureus* at the water-to-land metamorphic transition [11], PaT-2 displays structural features consistent with free-radical scavenging, and cell-based assays confirmed that it attenuates the pharmacologically induced overproduction of reactive oxygen and nitrogen species in microglial and neuroblast cells [11]. These findings establish PaT-2 as a biologically active antioxidant peptide. However, whether and how PaT-2 modulates the endogenous antioxidant machinery—particularly the glutathione system, which serves as the primary redox buffer in mammalian cells—remains unknown.

In this context, the present study pursued two complementary aims. First, we provide a preliminary histological and histochemical characterization of the skin of *Pithecopus oreades* and compare it with *P. azureus*, incorporating MALDI mass spectrometry imaging (MALDI-MSI) to map the spatial distribution of PaT-2 across body regions in both species. Second, we extend the functional characterization of PaT-2 by assessing its effects on glutathione status—specifically total glutathione (tGSH), reduced glutathione (GSH), oxidized glutathione (GSSG), and the GSSG/tGSH ratio—in BV2 microglial cells subjected to menadione-induced oxidative stress. Together, these findings advance the integumentary biology of *Pithecopus*, suggest differences in peptide distribution between the specimens examined, and provide mechanistic insight into PaT-2 antioxidant action at the level of cellular redox metabolism.

## 2. Materials and Methods

### 2.1. Study Area and Animal Collection

Specimens were collected at Fazenda Água Limpa (FAL) of the University of Brasília, Federal District, Brazil—part of the Environmental Protection Area Gama Cabeça de Veado (~4500 hectares of Cerrado). Collections were carried out on March 1st, 7th, and 14th and 26 October 2024, during the rainy season, beginning at 8:00 p.m. Animals were located by conducting active searches along watercourses, artificial ponds (coordinates: 15°57′41.1″ S 47°58′09.2″ W; 15°56′18.7″ S 47°56′29.7″ W), and upland streams (15°58′39.1″ S 47°56′44.0″W). Six individuals were collected—three *Pithecopus oreades* and three *P. azureus*—under IBAMA license no. 31066-1/SISBIO. Four individuals (two per species) were used for histological and histochemical analyses; two individuals (one per species) were used for MALDI imaging mass spectrometry.

### 2.2. Histology and Histochemistry

Animals were euthanized by immersion in 2% aqueous lidocaine hydrochloride or by direct intracranial injection, in accordance with Normative Resolution No. 37/2018 of the National Council for the Control of Animal Experimentation. After confirmation of death, skin was dissected from five body regions: head, forelimbs, dorsum, inguinal area, and hind limbs. Tissues were fixed in Carnoy’s solution and processed for paraffin embedding (dehydration, clearing in coconut oil, three paraffin infiltration steps). Sections of 5 µm were cut with a rotary microtome (Leica 2125RT, Wetzlar, Germany) and stained with hematoxylin and eosin (H&E), periodic acid–Schiff (PAS), and Alcian blue at pH 1.0 and pH 2.5. For each individual, eight sections per body region were examined, across eight body regions (head, neck, dorsum, forelimbs, inguinal area, and hindlimbs), totaling 64 sections per individual. Histological identification and measurement of gland types were not performed blind to species or body region, as slides were labeled with this information prior to analysis.

### 2.3. MALDI Mass Spectrometry Imaging

MALDI-MSI [12] was used to map the distribution of PaT-2 in the tegument of specimens, as previously performed in anurans [11,13]. Dissected skin fragments were placed on glass slides, allowed to air-dry, and transferred to a MALDI target plate using electrically conductive double-sided adhesive tape (3M). A matrix solution of α-cyano-4-hydroxycinnamic acid (CHCA; 50% acetonitrile, 40% Milli-Q water, 10% trifluoroacetic acid, *v*/*v*/*v*) was deposited onto the tissue surface and allowed to dry. Acquisitions were performed on an MALDI-TOF AutoFlex Speed mass spectrometer (Bruker Daltonics, Bremer, Germany) in positive reflective mode with constant laser intensity and 1 mm spatial resolution. No specific criteria were applied to select regions of interest; the entire dissectible tissue area obtained from each body region was scanned. The instrument was calibrated using a calibration solution deposited onto a skin tissue fragment. No quantitative normalization was applied to allow comparison of absolute ion intensities across specimens or tissue sections; each ion density map was independently rescaled to its own maximum intensity for display purposes only. Data acquisition and analysis were performed with FlexImaging 4.0 software.

The ion at *m*/*z* 696.4 was assigned to the [M+K]^+^ adduct of PaT-2, a peptide first described by us in *P. azureus* [11], on the basis of (i) prior knowledge of the species and identification of this peptide and its characteristic ionization behavior in *P. azureus* skin secretion by MALDI-MSI [11]; (ii) LC-MS analysis of the crude skin secretion, in which the extracted-ion chromatogram showed a single dominant signal (Figure S1A) with accurate-mass and adduct matching to the [M+H]^+^, [M+Na]^+^, and [M+K]^+^ adducts of PaT-2 (Figure S1B), and MS/MS of the *m*/*z* 696.4 ion did not yield informative fragment ions (Figure S1C), consistent with the limited fragmentation previously reported for adducts of this type [14]; and (iii) MALDI-MS analysis of the synthetic peptide standard, which reproduced the same [M+K]^+^ adduct (Figure S1D).

### 2.4. Peptide Synthesis and Characterization

PaT-2 (FPPWL-NH_2_) was synthesized by Fmoc solid-phase peptide synthesis (SPPS) and purified by reverse-phase HPLC (RP-HPLC) as previously described [11]. Briefly, Fmoc-protected amino acid derivatives (Fmoc-Phe-OH, Fmoc-Pro-OH, Fmoc-Trp(Boc)-OH, and Fmoc-Leu-OH) were coupled using DIC and Oxyma Pure, with Fmoc deprotection by 20% 4-methylpiperidine in DMF. Cleavage from the resin was performed with a TFA-based cocktail (TFA, thioanisole, water, phenol, ethanedithiol) for 90 min. The crude peptide was precipitated with isopropyl ether and lyophilized. Purification was carried out on a C18 column (250 × 10 mm, 5 µm) in Shimadzu HPLC system (Kyoto, Japan) with an acetonitrile gradient (5–95% over 60 min) at 10 mL/min, with UV detection at 216 and 280 nm. Peptide identity was confirmed by LC-MS and LC-MS/MS in positive ion mode using the Information Dependent Acquisition (IDA) method; spectra were analyzed with PeakView v2.1 and the Interactive Peptide Spectral Annotator online tool.

### 2.5. Cell Culture and Determination of Menadione Working Concentration

BV2 microglial cells (*Mus musculus*, BCRJ #0356) were cultured in DMEM supplemented with 10% fetal bovine serum (FBS, Gibco, Thermo Fisher Scientific, Waltham, MA, USA), 1% streptomycin–penicillin, and 1% MEM non-essential amino acids, at 37 °C in a humidified 5% CO_2_ atmosphere. Cells were used between passages 18 and 21. To select a working concentration of menadione for oxidative stress induction, cell viability was assessed by the resazurin assay (10 µg/mL, 4 h incubation) in 96-well plates (5000 cells/well) after 24 h treatment across eight concentrations (1.56–200 µM; 6 wells per concentration). The half-maximal cytotoxic concentration (CC_50_) was estimated by fitting a four-parameter log-logistic model to the dose–response data in R, with 95% confidence intervals calculated by the delta method. This experiment was repeated independently once.

### 2.6. Glutathione Status Assay

In a first assay, BV2 cells (1 × 10^6^/well in 6-well plates) were treated for 24 h with 10 µM menadione or DMEM (control). Each treatment was performed in three replicate wells, and the assay was independently repeated twice, resulting in a total of three independent experiments. In a second assay, cells were subjected to four conditions: control (DMEM), 10 µM menadione, 50 µM PaT-2, and 50 µM PaT-2 combined with 10 µM menadione. Each of the four treatments was performed in three replicate wells, and the assay was independently repeated twice, resulting in a total of three independent experiments. In both assays, cells were detached after treatment, washed with PBS, and assayed for total glutathione (tGSH), reduced glutathione (GSH), and glutathione disulfide (GSSG) by the enzymatic recycling method [15,16]. The GSSG/tGSH ratio was calculated as an index of cellular redox status. Glutathione values were normalized to total protein content determined by the Bradford assay [17].

### 2.7. Statistical Analysis

All statistical analyses were performed in RStudio (v2026.05.1+225, [18]. For the first glutathione assay (two groups), differences between control and menadione-treated cells were assessed by Student’s *t*-test or equivalent Welch’s *t*-test when F-test revealed a violation of the variance homogeneity assumption. For the second glutathione assay (four groups), data were first tested for normality by the Shapiro–Wilk test; normally distributed variables were compared by one-way ANOVA followed by Tukey’s HSD post hoc test, while non-normally distributed variables were compared by the Kruskal–Wallis test followed by Dunn’s post hoc test. Statistical significance was set at *p* < 0.05.

## 3. Results

### 3.1. Histological Analysis of the Skin of P. azureus and P. oreades

**Figure 2:**
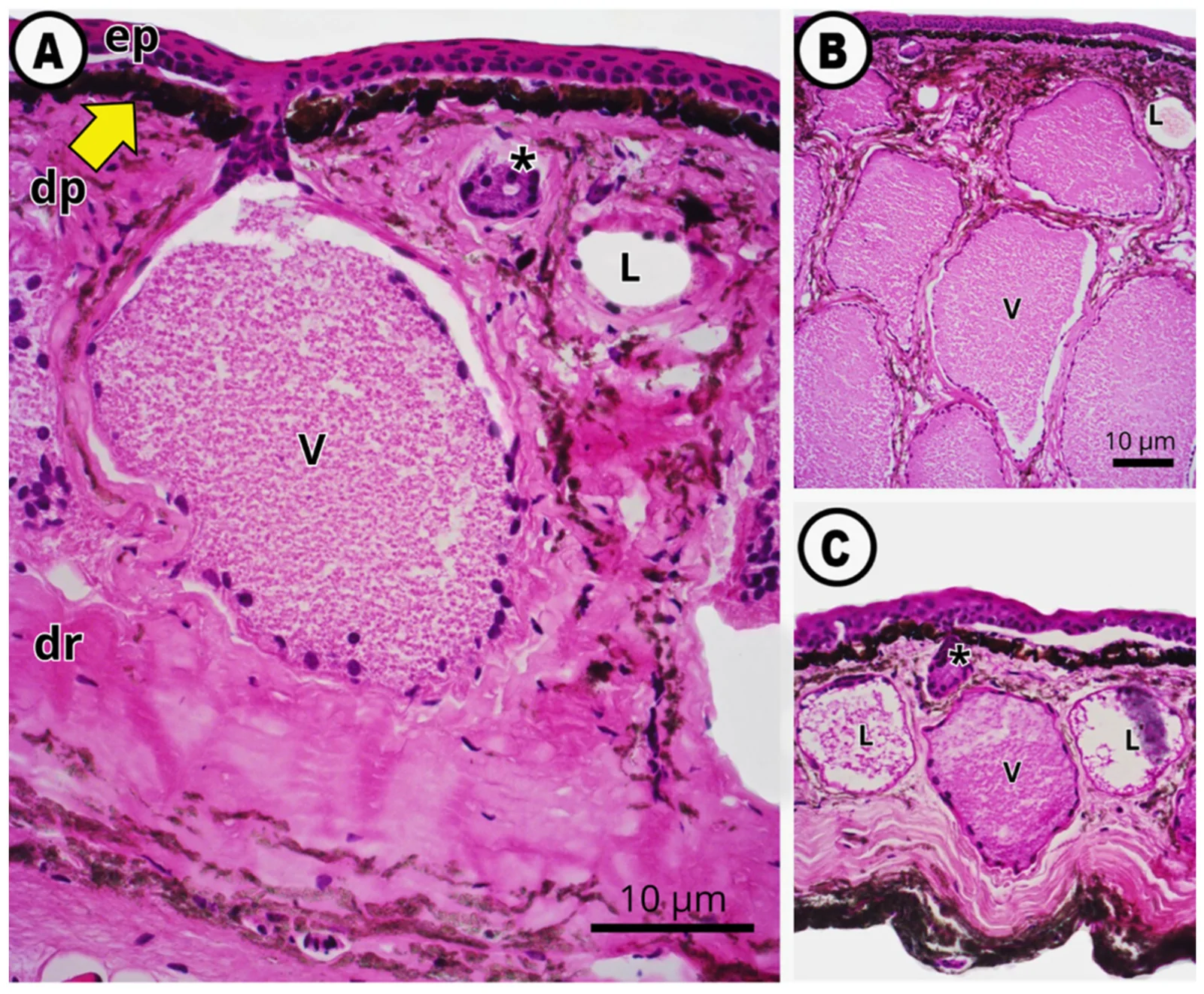
Morphological characterization of the skin of *P. oreades*. (**A**) Head region showing the epidermis (ep), papillary dermis (dp), and reticular dermis (dr). In the papillary dermis, chromatophores (arrow) and mucous glands (*) are visible. In the reticular dermis, lipid-rich granular glands (L) and serous granular glands (V, venomous) are observed. (**B**) Neck region, showing many serous granular glands (V). (**C**) Dorsal region, showing thinner skin with smaller and more sparsely distributed serous granular glands (V). Scale bar = 10 µm. Staining method: H&E (*n* = 2 individuals per species; 8 sections examined per body region, 64 sections per individual in total; representative sections shown).

In both species, the skin is organized into an epidermis—comprising basal cells, keratinocytes, and a superficial keratinized layer—and a dermis subdivided into two distinct layers. The papillary dermis consists of loose connective tissue containing chromatophores and small glands, while the deeper reticular dermis is composed of dense irregular connective tissue with collagen fibers and larger glands (Figure 2A). All gland types connect to the outermost epidermal layer via secretory ducts.

**Figure 3:**
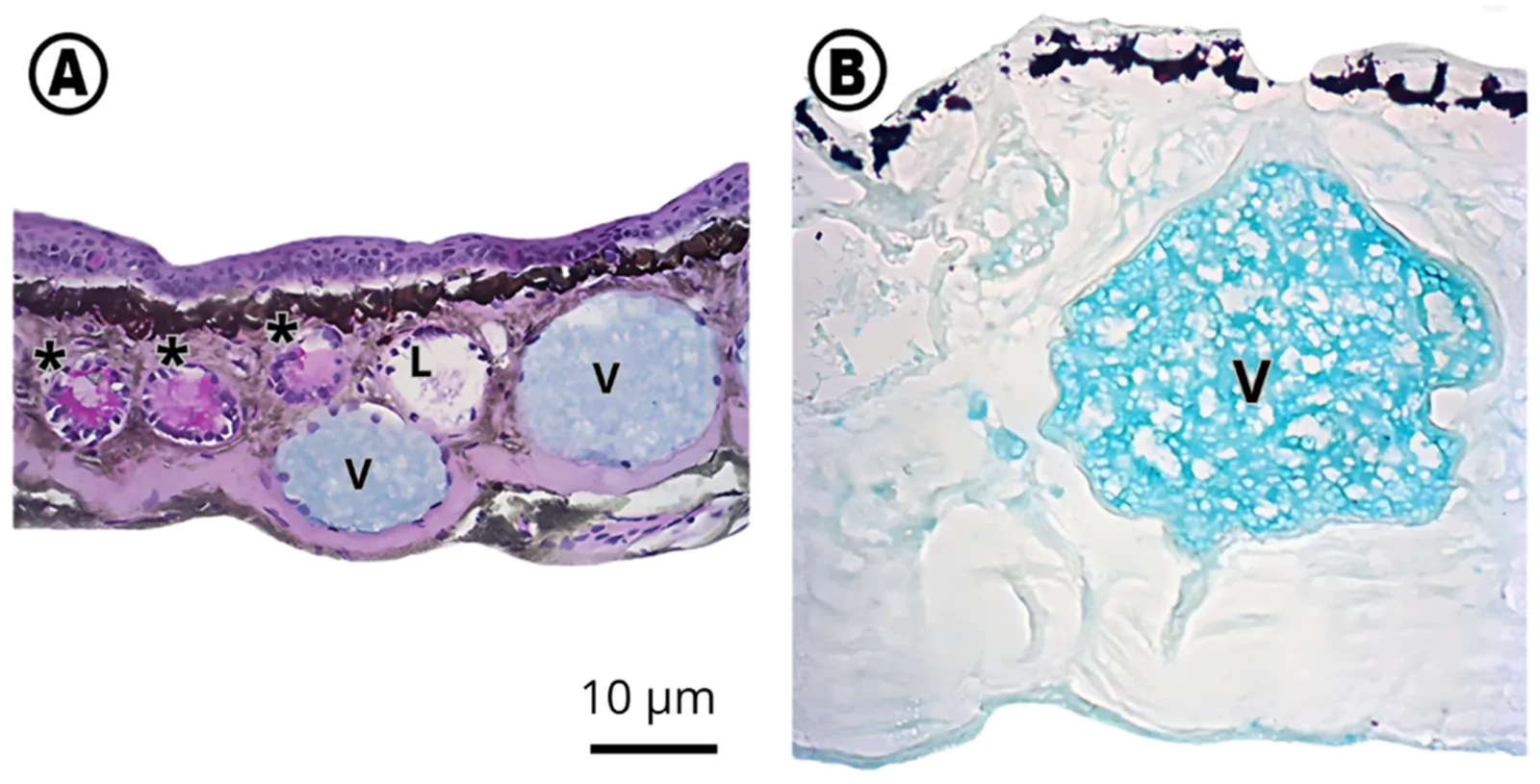
Histochemical characterization of the skin of *P. oreades* and *P. azureus*. (**A**) Forelimb region showing mucous glands (*) positive for PAS, lipid-rich granular glands (L) weakly positive for PAS, and serous granular (venom) glands (V) positive for Alcian blue pH 2.5. (**B**) Inguinal region showing serous granular (venom) gland (V) positive for Alcian blue pH 1.0. Scale bar = 10 µm. Staining methods used: Alcian Blue pH 2.5 + PAS (**A**) and Alcian Blue pH 1.0 (**B**). *n* = 2 individuals per species; 8 sections examined per body region, 64 sections per individual in total; representative sections shown.

Three types of glands were identified throughout the dermis based on their morphology and histochemical reactivity: mucous glands, lipid-rich granular glands, and serous granular glands (Figure 2 and Figure 3).

Mucous glands were located in the papillary dermis and exhibited strong PAS affinity, consistent with an abundance of neutral mucopolysaccharides (Figure 3A). Lipid-rich granular glands were medium-sized and showed only a mild positive reaction to PAS (Figure 3A). Serous granular glands (venom glands) varied markedly in size and number depending on body region: in the head (Figure 2A) and neck (Figure 2B) regions, they were larger and more numerous, displaying an alveolar appearance, whereas in the remaining body regions, including the dorsum (Figure 2C), they were smaller and more sparsely distributed. A thin layer of myoepithelial cells lines the margin of these glands. Alcian blue staining at pH 2.5 and pH 1.0 revealed the presence of carboxylated acidic polysaccharides and sulfated polysaccharides, respectively, within serous gland granules (Figure 3A,B).

**Figure 4:**
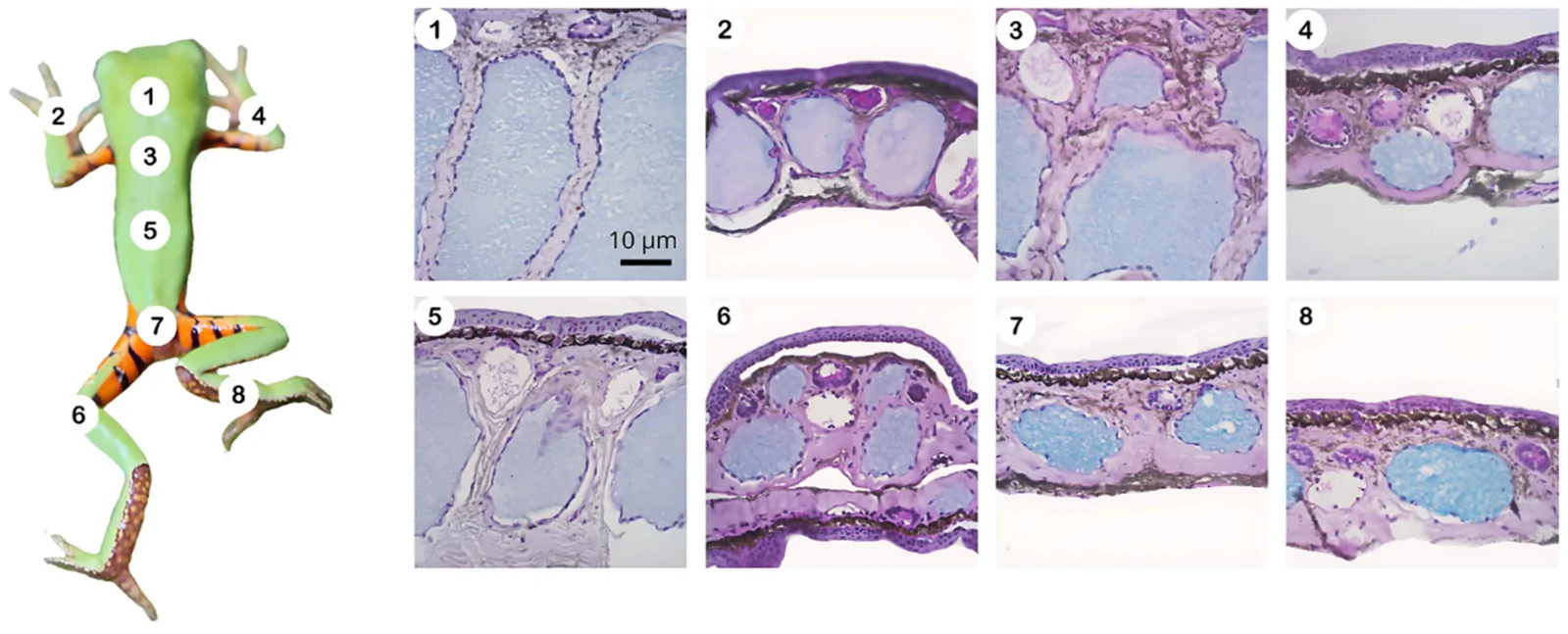
Distribution of glands in different body regions of *P. oreades*. Venom glands are more numerous and larger in the head (1), neck (3), and dorsal (5) regions. In other areas, such as the left (2) and right (4) forelimbs, inguinal region (7), and left (6) and right (8) hindlimbs, the glands are of medium size and more sparsely distributed. Staining method: Alcian Blue pH 2.5 + PAS (*n* = 2 individuals per species; 8 sections examined per body region, 64 sections per individual in total; representative sections shown).

Overall, the skin of *P. oreades* and *P. azureus* showed a similar histological organization, with serous glands being more abundant and larger in the cephalic, neck, and dorsal regions compared with the limbs (Figure 4).

### 3.2. MALDI Imaging

**Figure 5:**
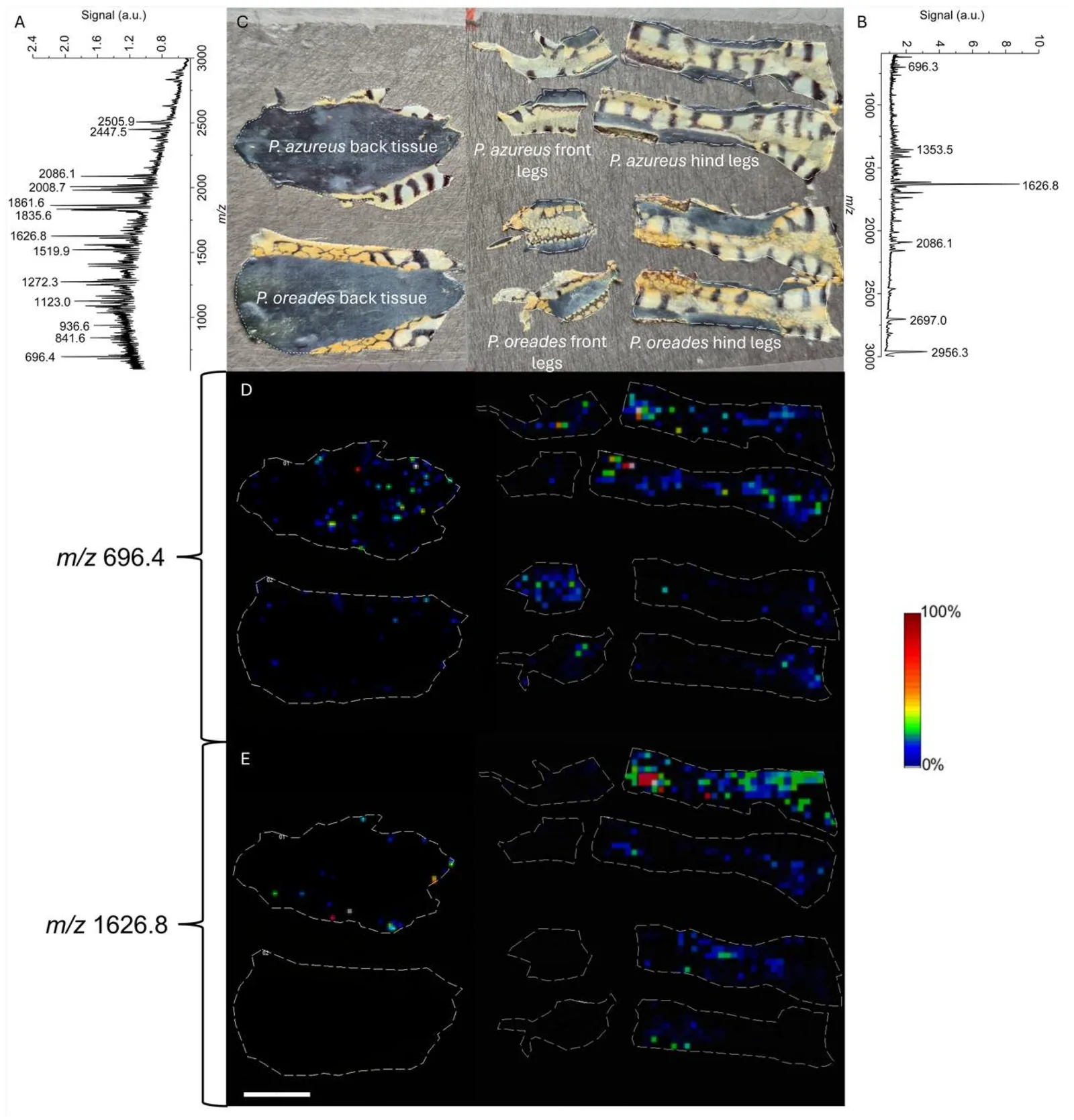
MALDI-MSI analysis of skin tissues from *P. azureus* and *P. oreades*. (**A**) Representative mass spectrum obtained from *P. azureus* skin tissues in the *m*/*z* 600–3000 range, highlighting the major detected ions. (**B**) Representative mass spectrum obtained from *P. oreades* skin tissues in the same mass range. (**C**) Dissected skin fragments mounted on the MALDI target plate, including dorsal skin, forelimbs, and hind limbs from both species. (**D**) Ion density maps corresponding to PaT-2 detected as its [M+K]^+^ adduct (*m*/*z* 696.4), showing heterogeneous spatial distribution and higher relative abundance in dorsal and limb tissues, especially in *P. azureus*. (**E**) Ion density maps corresponding to *m*/*z* 1626.8, assigned to a peptide previously reported in *Pithecopus* spp. and assigned to the sequence IPHAINAVSAIAKHF-NH_2_, a truncated homolog of phylloseptin PS-6 (Figure S2). The ion was predominantly localized in limb regions. Color scale indicates relative signal intensity, independently rescaled for each ion map from low (black, 0%) to high (red, 100%) for display purposes; absolute intensities were not normalized for comparison across specimens. Scale bar = 10 mm. *n* = 1 individual per species; ion density maps represent a single specimen.

MALDI-MSI analysis of skin tissues from *P. azureus* and *P. oreades* yielded average mass spectra in the *m*/*z* 600–3000 range with multiple detectable ions, including signals consistent with peptides previously reported in *Pithecopus* species (Figure 5A,B).

The ion at *m*/*z* 696.4, corresponding to the [M+K]^+^ adduct of PaT-2 ([M+H]^+^ = 658.4 Da, see Figure S1), was detected in the skin of both species but showed a heterogeneous spatial distribution across the integument (Figure 5D). In the specimens examined, this ion was predominantly localized in dorsal and limb tissues in both species; however, relative signal intensity appeared higher in the *P. azureus* individual than in the *P. oreades* individual analyzed. As MALDI-MSI was performed on a single specimen per species, this observation should be interpreted as preliminary rather than a confirmed interspecific difference.

A second ion, at *m*/*z* 1626.8, assigned to the sequence IPHAINAVSAIAKHF-NH_2_, a truncated homolog of the antimicrobial peptide phylloseptin PS-6 [19], showed a distinct spatial pattern, with predominant localization in limb regions and comparatively lower abundance in dorsal tissues (Figure 5E). This identity was confirmed by MS/MS fragmentation of the [M+H]^+^ ion (*m*/*z* 1587.9) (Figure S2); and the presence of the [M+K]^+^ adduct (*m*/*z* 1626.8) tracked in the MALDI-MSI.

Together, these results indicate that peptide-associated ions are non-uniformly distributed across the integument in the specimens examined, with the *P. azureus* individual showing higher PaT-2 abundance than the *P. oreades* individual analyzed.

### 3.3. Menadione-Induced Oxidative Stress and the Effect of PaT-2 on Glutathione Status

To select an appropriate concentration of menadione for redox imbalance induction, we assessed its cytotoxic profile in BV2 microglial cells using the resazurin assay. CC_50_ values from two independent experiments were 9.46 µM (95% CI: 8.55–10.36 µM) and 13 µM (95% CI: 10.21–15.79 µM), yielding a mean of approximately 11 µM. A concentration of 10 µM was therefore selected for subsequent experiments as a dose expected to induce measurable oxidative stress while preserving cell viability.

**Figure 6:**
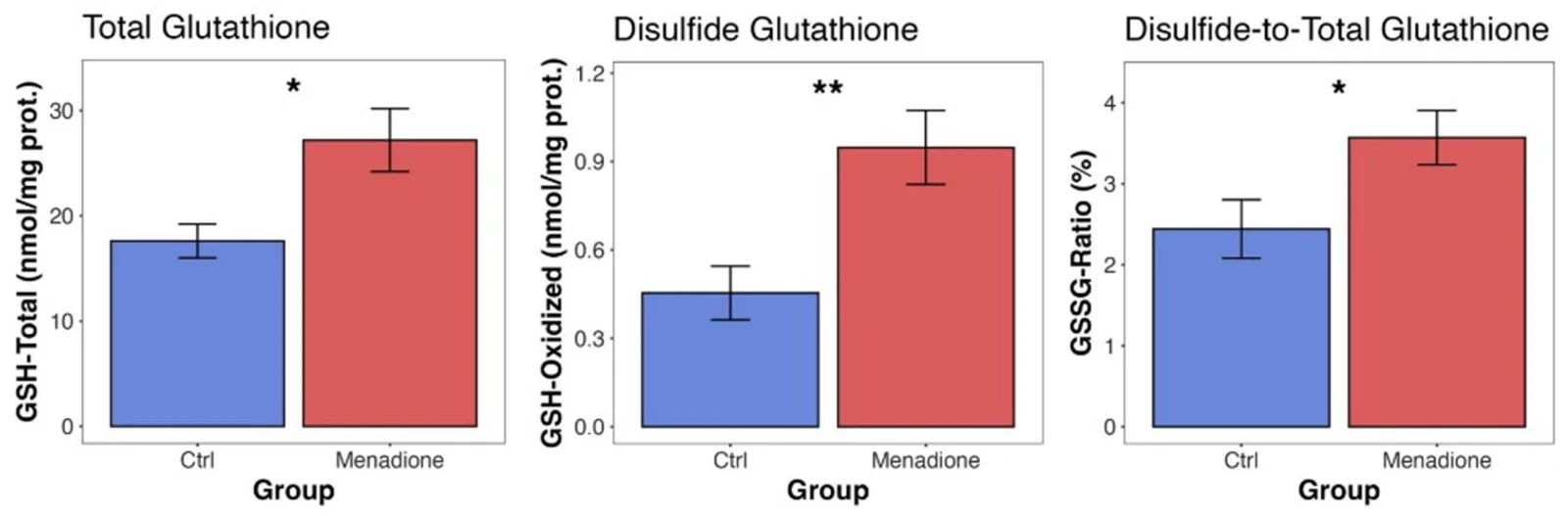
Glutathione status in BV2 cells after treatment with menadione (10 µM). The figure presents total glutathione (tGSH), oxidized glutathione (GSH-Oxidized), and the GSSG/tGSH ratio (GSSG-Ratio). Control cells are represented in blue, while cells treated with menadione (10 µM) are shown in red. Statistical significance is indicated by asterisks (*p* < 0.05 = “*”; *p* < 0.01 = “**”). Bars represent mean ± SEM. *n* = 3 independent experiments. *t*-test: tGSH, *p* = 0.0269; GSH-Oxidized, *p* = 0.00297; GSSG-Ratio, *p* = 0.0402.

We first characterized the effect of 10 µM menadione on glutathione status in BV2 cells. After 24 h of treatment, GSSG levels increased by 124% (from 0.487 to 1.09 nmol/mg protein) and the GSSG/tGSH ratio shifted from 2.54% to 3.78% (a 49% increase), indicating a more oxidized redox state. Concurrently, GSH and tGSH also increased by 53% and 54%, respectively (from 19.2 to 29.3 and from 19.7 to 30.4 nmol/mg protein), consistent with a compensatory increase in glutathione levels in response to oxidative challenge; whether this reflects increased glutathione synthesis specifically was not directly tested (Figure 6).

**Figure 7:**
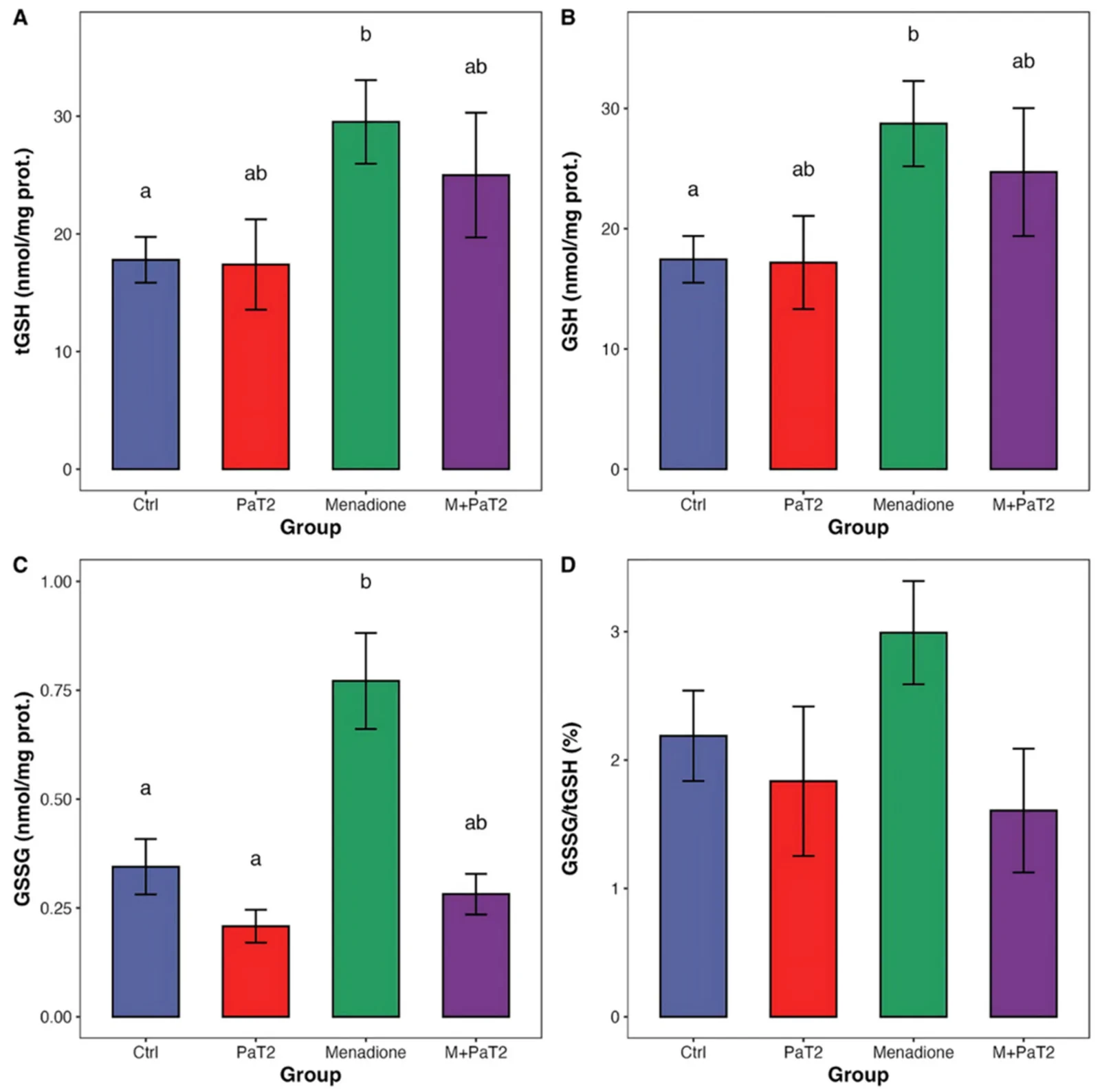
Effects of PaT-2 on glutathione-related parameters in menadione-treated cells (BV2). (**A**) Total glutathione (tGSH), (**B**) reduced glutathione (GSH), (**C**) oxidized glutathione (GSSG), and (**D**) GSSG/tGSH ratio. Groups analyzed: control (Ctrl), cells treated with PaT-2, cells treated with menadione, and cells treated with menadione + PaT-2 (M+PaT-2). Bars represent mean ± SEM. Groups not sharing a letter are significantly different; groups sharing a letter are not. *n* = 3 independent experiments.

To evaluate the effect of PaT-2 on these parameters, cells were subjected to four conditions: control (DMEM), 10 µM menadione, 50 µM PaT-2, and 50 µM PaT-2 combined with 10 µM menadione. PaT-2 alone did not alter any glutathione parameter relative to the control, confirming its lack of intrinsic cytotoxicity. In the menadione-only group, results were consistent with the first experiment. In cells co-treated with PaT-2 and menadione, GSSG levels were numerically lower relative to the menadione-only group, indicating attenuation of glutathione oxidation (Figure 7).

Statistical analysis by the Kruskal–Wallis test revealed significant group effects for GSH (*p* = 0.019), GSSG (*p* = 0.001), and tGSH (*p* = 0.014), but not for the GSSG/tGSH ratio (*p* = 0.181). Post hoc comparisons (Dunn’s test) showed that menadione-treated cells had significantly higher GSH (p_adj_ = 0.036) and GSSG (p_adj_ = 0.012) than controls, and higher GSSG than PaT-2-treated cells (p_adj_ = 0.003). Total glutathione (tGSH) was also significantly elevated in the menadione group relative to controls (p_adj_ = 0.027).

## 4. Discussion

The skin of *Pithecopus oreades* and *P. azureus* shows the conserved histological organization typical of anurans, with an epidermis overlying a dermis rich in mucous and granular glands [4]. This shared architecture across phylogenetically distant taxa underscores the importance of cutaneous secretions as multifunctional systems in amphibian physiology, simultaneously enabling hydration, chemical defense, and antimicrobial protection.

In both species, serous glands were markedly larger and more abundant in the dorsal and cephalic regions than in the limbs—a pattern consistent with reports in other anurans, including *Litoria caerulea* [20] and *Telmatobius coleus* [21], where dorsal dermal thickness correlates with greater gland density and size. In *Pithecopus*, this regional specialization likely reflects evolutionary pressure for enhanced chemical defense in the areas most exposed to predator contact and environmental stress [22]. The behavioral observations of Barbeau & Lillywhite [23]—who described coordinated limb-rubbing across the dorsal surface, head, and inguinal regions in arboreal frogs—add a functional dimension to this morphological pattern: such grooming behaviors promote glandular discharge and redistribution of secretions across the integument, potentially optimizing chemical defense under dehydration or predator pressure.

Histochemical analyses confirmed the chemical complexity of these secretions. Mucous glands reacted strongly to PAS staining, consistent with prior reports in other amphibians [20] and reflecting an abundance of glycoproteins and neutral mucopolysaccharides that maintain hydration and cutaneous adhesion [24]. Alcian blue staining at pH 2.5 and pH 1.0 revealed carboxylated acidic polysaccharides and sulfated polysaccharides, respectively, within serous gland granules [25]. The identification of lipid-rich granular glands in the papillary dermis—comparable to structures described in arboreal Hylidae [23]—suggests an additional barrier function that may reduce evaporative water loss, further reinforcing the multifunctional nature of the amphibian integument.

The larger, more numerous serous glands observed in the dorsal region are consistent with reports in other terrestrial/fossorial anurans, where greater dermal thickness and gland size in dorsal skin appear to be a conserved pattern across species, related to mechanical protection needs [21]. However, this conserved glandular organization across anuran species contrasts with the substantial diversity reported in the biochemical composition of their secretions, which appears to be the primary axis of interspecific variation rather than gross gland morphology [26]. Furthermore, O’Donohoe et al. [27] showed that granular glands in neotropical odontophrynid frogs are not exclusively defensive: PAS-positive neutral glycoconjugates within these glands suggest an additional role in cutaneous hydration and water balance. In this light, the pronounced development of serous glands in the dorsal region of *Pithecopus* may reflect not only increased capacity for storage of bioactive peptides such as PaT-2, but also an adaptation supporting skin protection and hydration in a body region subject to greater environmental exposure in these arboreal species.

MALDI-MSI provided a spatially resolved view of the peptide complement across the integument of both species, demonstrating that bioactive molecules are not uniformly distributed over the skin surface. The [M+K]^+^ ion of PaT-2 (*m*/*z* 696.4) showed a heterogeneous distribution, with higher relative abundance detected in the *P. azureus* specimen than in the *P. oreades* specimen, particularly in the dorsal skin. In contrast, limb regions showed a more complex and overlapping chemical profile between the two individuals. This difference is noteworthy because the two species share a broadly conserved skin architecture, as demonstrated by the histological and histochemical data of the present study. However, as MALDI-MSI was performed on a single individual per species, this observation should be regarded as preliminary rather than a confirmed interspecific pattern. If confirmed in larger samples, differential PaT-2 abundance would suggest that closely related species can display species-specific chemical phenotypes, potentially reflecting distinct ecological pressures, microhabitat exposure, or physiological requirements for cutaneous redox protection. The dorsal enrichment of PaT-2 in *P. azureus* is also consistent with the regional glandular specialization described above: the larger, more abundant serous glands in the dorsum are expected to contribute greater secretory output, potentially including antioxidant peptides relevant to the protection of this highly exposed surface. Thus, the regional distribution observed for PaT-2 is consistent with the anatomical organization of the skin, although matrix-related heterogeneity may also contribute to local variation in ion intensity. Taken together, these findings raise the possibility that peptide secretion is regulated at both the species and body-region level, adding a further layer of organization to the integumentary defense system; confirming this will require MALDI-MSI analysis of additional specimens per species.

A second ion, at *m*/*z* 1626.8—assigned to IPHAINAVSAIAKHF-NH_2_, a proposed truncated form of a phylloseptin-like antimicrobial peptide previously reported in *Pithecopus*—showed predominant localization in limb regions, partially overlapping with the distribution of PaT-2. This ion’s identity as IPHAINAVSAIAKHF-NH_2_, a truncated homolog of phylloseptin PS-6, was confirmed by MS/MS analysis (Figure S2). Its spatial co-occurrence with PaT-2 is consistent with amphibian skin defense integrating multiple molecular functions: antimicrobial peptides limit microbial proliferation, while antioxidant peptides may reduce the oxidative damage associated with environmental stress, inflammation, or microbial challenge. PaT-2 may therefore function as part of a broader cutaneous defense network rather than as an isolated antioxidant molecule.

The functional data provide a mechanistic complement to these spatial observations. Menadione is an established ROS-inducing stressor that operates by redox cycling [28,29], and the concentration of 10 µM—near the experimentally determined CC_50_—was selected to induce measurable oxidative stress while preserving cell viability. The simultaneous increase in GSSG, GSH, and tGSH following menadione treatment is consistent with a two-phase cellular response: an initial oxidation of the glutathione pool—reflected in elevated GSSG and the GSSG/tGSH ratio, a widely used index of cellular redox status [30,31,32]—followed by a compensatory upregulation of glutathione biosynthesis [33]. Glutathione is the principal low-molecular-weight thiol antioxidant in mammalian cells [34], operating as a redox buffer through the coupled action of peroxidases, reductases and transferases [35,36,37]; its depletion compromises cellular integrity and is associated with increased susceptibility to oxidative injury [38,39,40,41].

PaT-2 at 50 µM showed no intrinsic cytotoxicity and, when co-administered with menadione, attenuated the accumulation of GSSG and the compensatory increase in glutathione synthesis. These results build on earlier work by Barbosa et al. [11], who demonstrated that PaT-2 reduces pharmacologically induced ROS and RNS overproduction in microglial and neuroblast cells, and extend those findings by showing that the peptide also modulates the endogenous antioxidant response at the level of glutathione status. Mechanistically, the aromatic residues of PaT-2—particularly tryptophan—can neutralize free radicals through hydrogen or electron donation [42], although whether the effect on glutathione is direct or mediated through modulation of biosynthetic enzymes or other redox signaling pathways remains to be determined.

Comparable modulation of glutathione handling by bioactive peptides has been reported in other cell models, although the underlying strategies differ. Consistent with our observation of menadione-induced increases in glutathione levels in BV2 cells, menadione exposure similarly increases glutathione levels via induction of γ-glutamylcysteine synthetase activity in Chinese hamster V79 cells [43], supporting the generality of this compensatory response across cell types. Indeed, in V79 cells [43] and other models [44], menadione first depletes GSH levels within short exposure times, indicating that the direction of the glutathione response to menadione is time-dependent, starting with depletion that is later compensated. Antioxidant peptides themselves also appear to act through diverse, non-uniform mechanisms on the glutathione pool. For example, calf thymic peptides increased glutathione levels and glutathione reductase activity in bovine pulmonary artery endothelial cells [45], whereas short antioxidant tetrapeptides produced peptide-specific, and in some cases opposite, effects on glutathione content in human K562 cells [46].

The present study assessed glutathione pool composition (tGSH, GSH, GSSG) but did not directly evaluate upstream antioxidant signaling pathways or enzymatic activities, such as Nrf2/HO-1 signaling, glutathione peroxidase, glutathione reductase, or glutamate–cysteine ligase catalytic subunit (GCLC). Future studies incorporating these targets will be important to clarify the molecular mechanism underlying the effect of PaT-2 on cellular glutathione status.

Taken together, these findings position PaT-2 as a functionally significant component of the *Pithecopus* chemical secretome, complementing antimicrobial peptides such as dermaseptins in a multifaceted cutaneous defense system. From a translational perspective, the non-cytotoxicity and redox-modulatory activity of PaT-2 in a single immortalized murine microglial cell line (BV2) support its further investigation as a candidate molecule for research into neurodegeneration and neuroinflammation, pathologies in which glutathione dysregulation and oxidative stress play central roles [47,48,49,50]. Translation toward therapeutic application would require additional studies addressing blood–brain barrier penetration, peptide stability, pharmacokinetics, and efficacy in vivo, none of which were assessed in the present study.

A limitation of this study is the small number of specimens examined—with two individuals per species processed for histology and histochemistry, and a single individual per species used for MALDI-MSI. While the histological and histochemical findings were consistent between the two individuals analyzed per species, the comparison of PaT-2 abundance between *P. azureus* and *P. oreades* by MALDI-MSI is based on a single specimen per species and should therefore be regarded as a preliminary, specimen-level observation rather than a confirmed interspecific difference. Studies with larger sample sizes will be needed to determine whether the pattern observed here reflects a consistent species-level difference in PaT-2 abundance.

## 5. Conclusions

This study provides a first histological description of *Pithecopus oreades* and confirms a conserved glandular organization in both species, with serous glands markedly enriched in the dorsal and cephalic regions. MALDI-MSI revealed that PaT-2 is distributed heterogeneously across the integument in the specimens examined, with higher abundance detected in the *P. azureus* individual than in the *P. oreades* individual despite their shared histological architecture, and partially overlapping with a putative phylloseptin-like ion; given the single-specimen sampling for this analysis, this interspecific comparison should be regarded as preliminary.

In BV2 microglial cells, PaT-2 co-treatment was associated with numerically lower menadione-induced GSSG accumulation and a smaller compensatory increase in glutathione levels, without cytotoxicity, extending previous evidence of ROS/RNS attenuation [11] to the level of endogenous glutathione status. These findings reinforce the ecological relevance of PaT-2 as a cutaneous antioxidant and support its further investigation as a candidate molecule for oxidative stress-related research, pending future in vivo and pharmacokinetic studies before any therapeutic application can be considered.

## Data Availability

The raw data supporting the conclusions of this article will be made available by the authors on request.

## Supplementary Materials

The following supporting information can be downloaded at https://www.mdpi.com/article/10.3390/antiox15091087/s1, Figure S1: Validation of the PaT-2 identity assignment (m/z 696.4, [M+K]+ adduct): (A) extracted ion chromatogram from LC-MS analysis of P. azureus skin secretion; (B) LC-MS mass spectrum of the corresponding secretion fraction; (C) LC-MS/MS fragmentation spectrum of the m/z 696.4 ion; (D) MALDI-MS analysis of synthetic PaT-2 peptide; Figure S2: Validation of the *m*/*z* 1626.8 ion identity (IPHAINAVSAIAKHF-NH_2_, a truncated homolog of phylloseptin PS-6): (A) MALDI-MS spectrum of the isolated skin secretion fraction; (B) MALDI-MS/MS fragmentation spectrum of the selected ion.
