# Supplementary material for: Skin Histology of Pithecopus spp. and Modulation of Glutathione Status by the Antioxidant Tryptophyllin PaT-2
Source: Antioxidants (Basel). 2026 Aug 29;15(9):1087. doi: 10.3390/antiox15091087 (PMC13603516; doi:10.3390/antiox15091087)
Supplement: Supplementary file 1 [file antioxidants-15-01087-s001.zip › Figure S1.pdf]

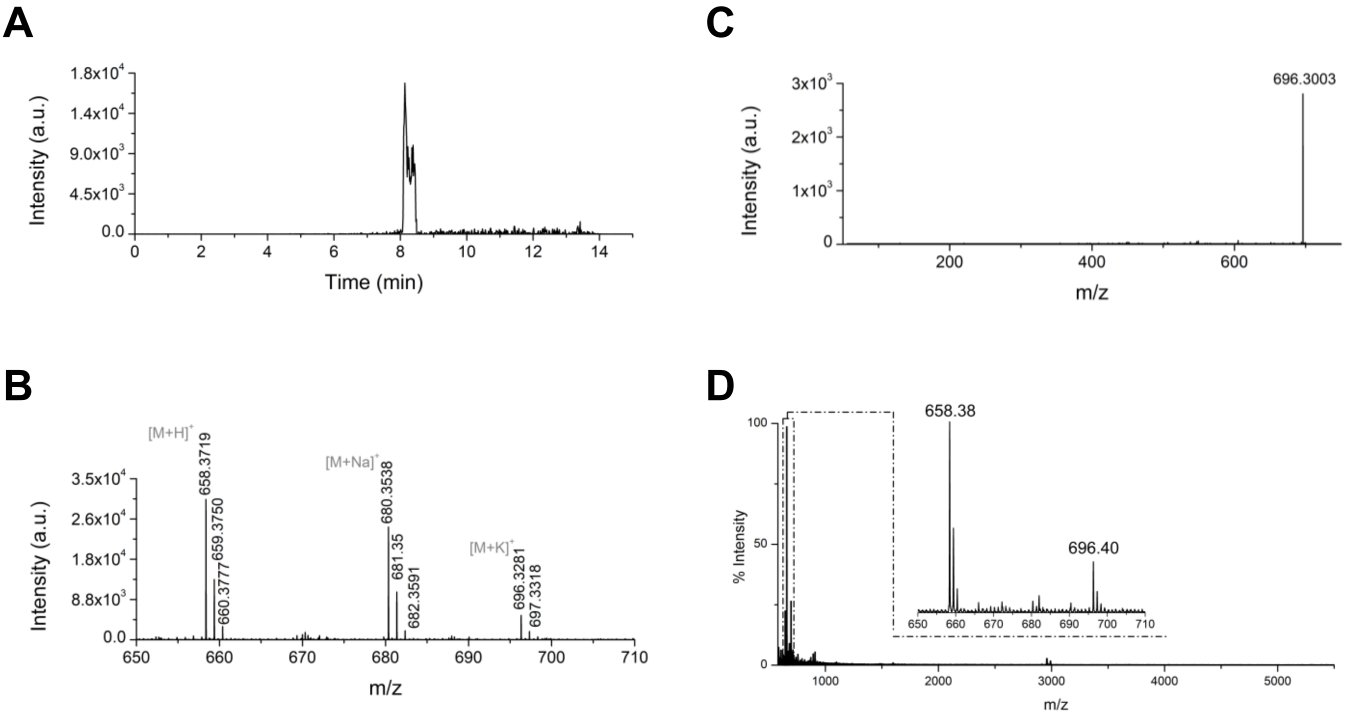
A
1.8x10^4
1.4x10^4
9.0x10^3
4.5x10^3
0.0
Intensity (a.u.)
0 2 4 6 8 10 12 14
Time (min)
B
[M+H]+
658.3719
659.3750
660.3777
[M+Na]+
680.3538
681.35
682.3591
[M+K]+
696.3281
697.3318
3.5x10^4
2.6x10^4
1.8x10^4
8.8x10^3
0.0
Intensity (a.u.)
650 660 670 680 690 700 710
m/z
C
696.3003
3x10^3
2x10^3
1x10^3
0
Intensity (a.u.)
200 400 600
m/z
D
658.38
696.40
100
50
0
% Intensity
650 660 670 680 690 700 710
1000 2000 3000 4000 5000
m/z
