# Supplementary material for: Skin Histology of Pithecopus spp. and Modulation of Glutathione Status by the Antioxidant Tryptophyllin PaT-2
Source: Antioxidants (Basel). 2026 Aug 29;15(9):1087. doi: 10.3390/antiox15091087 (PMC13603516; doi:10.3390/antiox15091087)
Supplement: Supplementary file 1 [file antioxidants-15-01087-s001.zip › Figure S2.pdf]

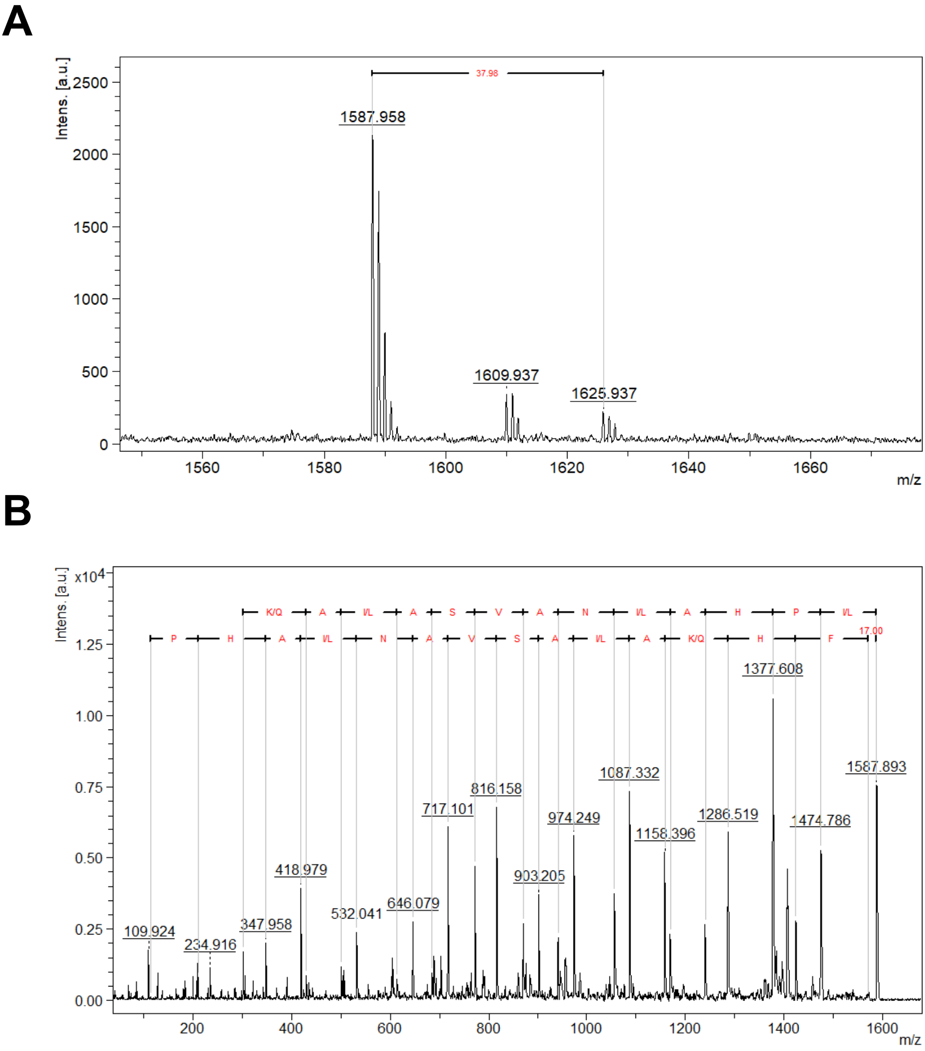
A
Intens. [a.u.]
2500
2000
1500
1000
500
0
37.98
1587.958
1609.937
1625.937
1560
1580
1600
1620
1640
1660
m/z
B
Intens. [a.u.]
x10^4
1.25
1.00
0.75
0.50
0.25
0.00
K/Q A I/L A S V A N I/L A H P I/L
P H A I/L N A V S A I/L A K/Q H F
17.00
109.924
234.916
347.958
418.979
532.041
646.079
717.101
816.158
903.205
974.249
1087.332
1158.396
1286.519
1377.608
1474.786
1587.893
200
400
600
800
1000
1200
1400
1600
m/z
